# Association of matrix metalloproteinases (MMPs) gene polymorphisms with periodontitis: a systematic review

**DOI:** 10.3205/dgkh000508

**Published:** 2024-10-23

**Authors:** Ruchi Pandey, Nandini Gupta, Tripti Jha, Tooba Bint E Manzoor

**Affiliations:** 1 Department of Periodontology, Manav Rachna Dental College, School of Dental Sciences, MRIIRS, Faridabad, Haryana, India; 2 Undergraduate student Manav Rachna Dental College, School of Dental Sciences, MRIIRS, Faridabad, Haryana, India

**Keywords:** chronic periodontitis, aggressive periodontitis, periodontitis, India, matrix- metalloproteinases, MMPs, single nucleotide polymorphisms, SNPs

## Abstract

Matrix metalloproteinases (MMPs) are proteinases released by gingival cells, macrophages and neutrophils, induced by potentially pathogenic periodontal bacteria of the subgingival plaque, which play a critical role in the pathogenesis of periodontal disease. The expression of MMPs is controlled by chromosome 11. Single nucleotide polymorphisms (SNPs) are linked with variations in the secretion of MMPs, resulting in periodontal disease progression. Genetic studies aim to find the markers for early diagnosis and prevention of the related diseases. This systematic review focuses on finding the association between the MMPs and periodontitis among Indians. A literature review was performed, including studies published between January 1^st^ 2012 and May 2024 were incorporated. This systematic review included 1,046 participants in seven Indian studies, and substantial evidence was found for an association between MMP-9 (–1562C/T) and periodontitis in Indian population.

## Abstract (de)

Matrix-Metalloproteinasen (MMPs) sind Proteinasen, die, induziert durch potentiell parodontal pathogene Bakterien der subgingivalen Plaque, von Gingivazellen, Makrophagen und Neutrophilen freigesetzt werden und eine entscheidende Rolle in der Pathogenese der Parodontitis spielen. Die Expression von MMPs wird durch das Chromosom 11 gesteuert. Einzelnukleotid-Polymorphismen (SNPs) werden mit Variationen in der Sekretion von MMPs in Verbindung gebracht, die zum Fortschreiten der Parodontalerkrankung führen. Genetische Studien zielen darauf ab, Entzündungs-Marker für die Frühdiagnose und Prävention zu finden. Die Übersichtsarbeit konzentriert sich auf den Zusammenhang zwischen MMPs und Parodontitis bei Indern. Es wurde eine Literaturrecherche durchgeführt, in die zwischen 2012 und Mai 2024 veröffentlichte Studien einbezogen wurden. Ausgewertet wurden sieben indische Studien (1.046 Teilnehmer). Es gab deutliche Hinweise auf einen Zusammenhang zwischen MMP-9 (–1562C/T) und Parodontitis in der indischen Bevölkerung.

## Introduction

Periodontal disease (PD) is one of the most common chronic ailments. It originates through the synergism of genes, environment, diet, oral health habits, tobacco use, etc. Initially, disease starts with gingivitis, progresses to the attachment apparatus of teeth, then leads to bone destruction and progressive attachment loss if left untreated [1].

The prevalence rate of periodontitis in Indian adults is 51%. A pervasiveness of 26.2% is seen for mild to moderate forms of periodontitis, whereas 19% are severe forms. The rate of severe periodontitis was higher in adults age 65 years or older. The 22.7% of India’s population residing in cities exhibited the highest pervasiveness for mild to moderate forms of periodontitis. Indian females (34.4%) had a lower rate of periodontitis than did males (42.2) [2].

Due to bacterial accumulation in dental plaques, interactions between the host and bacteria occur; these can lead to a breakdown of the proteinaceous extracellular matrix (ECM) consisting of collagen, matrix glycoproteins, proteoglycans, elastins, and gelatin [3]. 

Matrix metalloproteinases (MMPs) play a critical role in remodelling the periodontal tissue. MMPs are also termed extracellular proteinases [4].To date, 23 MMPs categorised into five types, have been documented in humans [5]. The majority of MMPs produced are in a pre-inflammatory latent form, and are transformed into active forms by serine proteases, plasmin, furin, and numerous other proteolytic enzymes. The proteolytic activity of MMPs starts once they are activated from the latent form; however, they can be naturally inhibited by tissue inhibitors of metalloproteinases (TIMPs) [6] and the natural inhibitors macroglobulins, as well as synthetic inhibitors, i.e., nonselective e.g. batimastat and BB-94 [7].

There is substantial evidence supporting the role of MMPs in periodontal tissue damage [8]. The gene controlling the expression of MMPs is located on chromosome 11. The excess production of MMPs disrupts homeostasis, causing ECM destruction. The expression of MMPs is increased in periodontal disease. Increased levels of MMP-1, MMP-2, MMP-3, MMP-8, and MMP-9 are present in gingival crevicular fluid (GCF), perimplant sulcular fluid (PISF) and gingival tissues during the diseased state [9]. The future of treatment lies in finding out the potential SNPs associated with the disease and targeting the same for treatment of the disease. Polymorphism can modify gene expression, leading to breakdown of periodontal tissues in PD [10], [11], [12]. Studies on the association of MMPs and periodontitis in Indians are scarce. Thus, the aim of the current systematic review was to elucidate associations between SNPs linked to MMPs and periodontitis among Indians.

## Material and methods

The current systematic analysis followed the PRISMA statement (preferred reporting items for systematic reviews and meta-analyses). The PICO criteria (population, intervention, comparison, outcomes) served as the basis for developing the focused question “Is there any association of MMPs and periodontitis in Indian population?” 

### Search method

**Figure 1:**
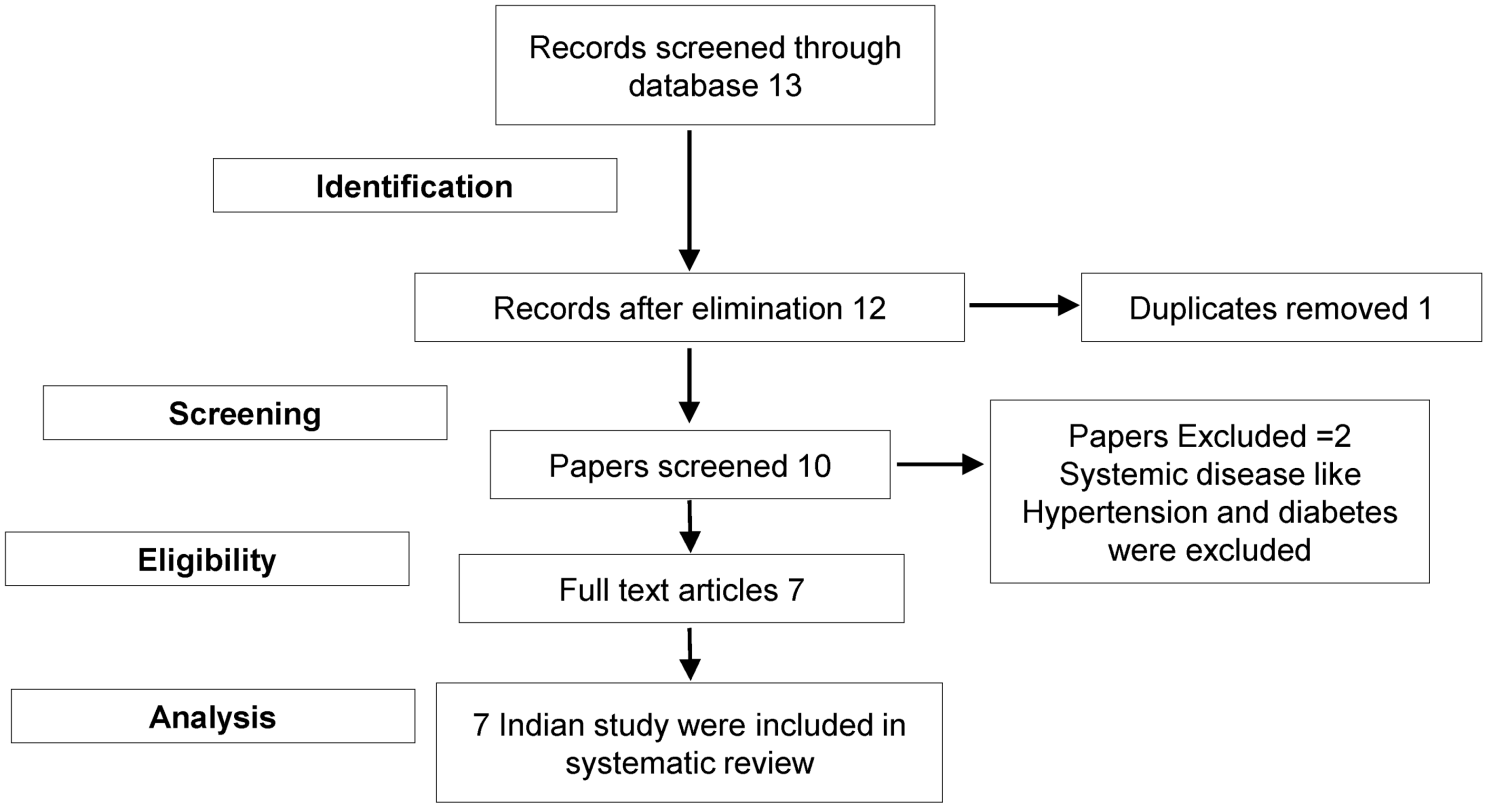
Consort diagram

Studies on the association of MMPs with periodontitis were searched in PubMed, Medline, Scopus and WoS databases using the search terms “Matrix Metalloproteinases polymorphism with Periodontitis” OR “Matrix Metalloproteinases polymorphism with Aggressive Periodontitis” OR “Matrix Metalloproteinase OR MMPs OR MMP and Polymorphism with chronic periodontitis”, “Single nucleotide Polymorphism AND Indian Population.” More databases were searched using similar and comparable words. Both digital and manual searches were conducted. The link between MMP polymorphisms and periodontitis susceptibility was examined by locating and including the full texts of relevant articles written in English (Figure 1 (Fig. 1)).

The studies included in this current systematic review were those that fount an association of MMPs and periodontitis, were conducted in an Indian population, were validated by polymerase chain reaction-restriction fragment length polymorphism (PCR-RFLP), and utilized a cross-sectional, experimental design. Studies conducted on mixed populations and those that failed to provide adequate information on genotype frequency or spread of genetic constitution for calculating relative ratios (ORs) and its discrepancies were excluded. 

## Results

After screening of 12 full length articles, 7 met the eligibility criteria and were found relevant as per inclusion and exclusion criterion. The characteristics of and conclusion drawn by all included studies are summarized in Table 1(Tab. 1).

After reviewing the seven studies on people of Indian origin and which met the inclusion criteria, evidence was found that periodontitis and MMP-9 (–1562C/T) are linked. MMP-8 (–799C/T) and MMP13 (–77A/G) can be associated with chronic forms of periodontitis in the Indian population.

## Discussion

Genetic differences and environmental variables are two elements that contribute to the complexity of periodontitis. AP and CP susceptibility is influenced by complex interrelationships between genetic makeup, phenotype, systemic diseases, oral-health habits and variation in lifestyle, e.g., factors concerning food and all forms of tobacco [13]. Polymorphism in genes influences the expression of MMPs, which are intimately linked to tissue remodelling processes in periodontitis, with excess production leading to the breakdown of extracellular matrix proteins [11]. There are numerous studies from different parts of the world establishing the causal role of SNPs in periodontal breakdown. However, genetic studies on the association of MMPs and periodontal disease in the Indian population are limited. Moreover, the studies have been carried out in different gene loci responsible for coding of different types of MMPs. Thus, the role of SNP associated with MMP and periodontitis cannot be clearly established. Another disparity is that the Indian population is highly diverse. Two general ethnicities exist; Dravidians and North Indians (Eurasians), so that any genetic study must focus on a diverse population sample to accurately identify SNPs that cause periodontal destruction in Indians [14].

MMP-9 have some positive association but more research based on diverse Indian population needs to be done, whereas the other two MMPs (MMP-8 and MMP-13) studied lack evidence.

## Notes

### Authors’ ORCIDs

- Ruchi Pandey: 0000-0001-9667-0778
- Nandini Gupta: 0000-0003-0086-3554
- Tripti Jha: 0009-0007-7842-8743
- ToobaBint E Manzoor: 0009-0003-2038-7599

### Funding

None. 

### Competing interests

The authors declare that they have no competing interests.

## Figures and Tables

**Table 1:**
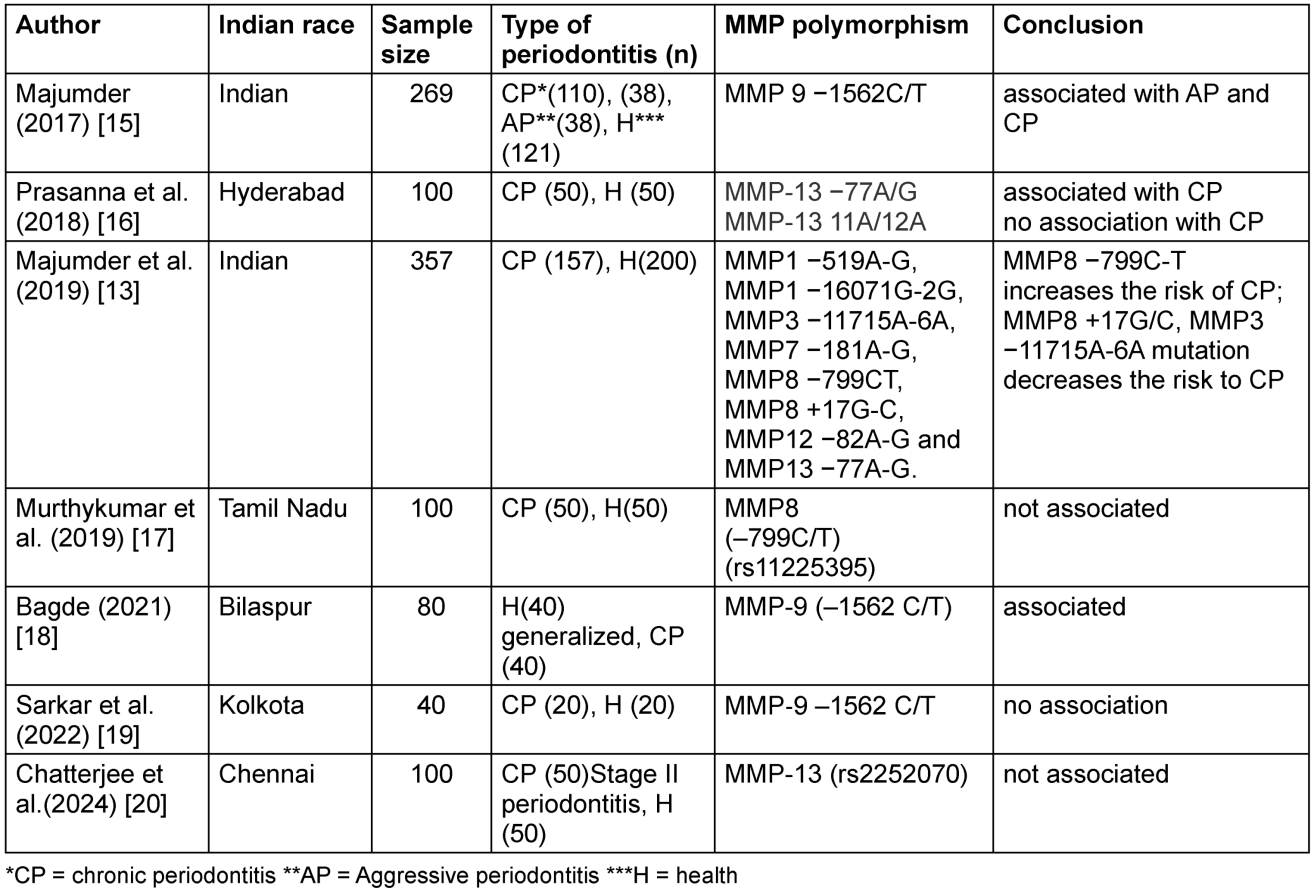
Demographics, sample sizes and SNPs of the included studies
